# Pan-Cancer Analysis Reveals Distinct Metabolic Reprogramming in Different Epithelial–Mesenchymal Transition Activity States

**DOI:** 10.3390/cancers13081778

**Published:** 2021-04-08

**Authors:** Ji-Yong Sung, Jae-Ho Cheong

**Affiliations:** 1 Department of Biomedical Systems Informatics, Yonsei University College of Medicine, Seoul 03722, Korea; jiyongsung@yuhs.ac; 2 Department of Surgery, Yonsei University College of Medicine, Seoul 03722, Korea; 3 Yonsei Biomedical Research Institute, Yonsei University College of Medicine, Seoul 03722, Korea; 4 Brain Korea 21 PLUS Project for Medical Science, Yonsei University College of Medicine, Seoul 03722, Korea; 5 Department of Biochemistry & Molecular Biology, Yonsei University College of Medicine, Seoul 03722, Korea

**Keywords:** epithelial–mesenchymal transition, metabolic reprogramming, tumor immune microenvironment, survival, energy metabolism

## Abstract

**Simple Summary:**

Recent genomic classification of tumors has stated that clinically refractory cancers aggregate as a distinct molecular subtype associated with epithelial–mesenchymal transition (EMT). EMT subtype tumors are clinically intractable due to shared malignant characteristics such as poor prognosis and metastasis and are resistant to chemotherapy and immune checkpoint blockades. Therefore, there is an urgent clinical need for the identification of potential therapeutic targets for this tumor subtype. Here, we profiled the metabolic signatures of 9452 samples across 31 cancer types based on EMT activity and identified that ~80 to 90% of cancer types had high carbohydrate and energy metabolism associated with the high EMT state. Furthermore, we identified *CHST14* as a potential metabolic target for the EMT subtype for stomach cancer associated with reprogramming of energy metabolism. Our analyses identified metabolic reprogramming associated with EMT, suggesting metabolism-associated targets for clinically refractory cancer subtypes.

**Abstract:**

Epithelial–mesenchymal transition (EMT) is critical for cancer development, invasion, and metastasis. Its activity influences metabolic reprogramming, tumor aggressiveness, and patient survival. Abnormal tumor metabolism has been identified as a cancer hallmark and is considered a potential therapeutic target. We profiled distinct metabolic signatures by EMT activity using data from 9452 transcriptomes across 31 different cancer types from The Cancer Genome Atlas. Our results demonstrated that ~80 to 90% of cancer types had high carbohydrate and energy metabolism, which were associated with the high EMT group. Notably, among the distinct EMT activities, metabolic reprogramming in different immune microenvironments was correlated with patient prognosis. Nine cancer types showed a significant difference in survival with the presence of high EMT activity. Stomach cancer showed elevated energy metabolism and was associated with an unfavorable prognosis (*p* < 0.0068) coupled with high expression of *CHST14*, indicating that it may serve as a potential drug target. Our analyses highlight the prevalence of cancer type-dependent EMT and metabolic reprogramming activities and identified metabolism-associated genes that may serve as potential therapeutic targets.

## 1. Introduction

Epithelial–mesenchymal transition (EMT) is defined as a change in the cellular organizational process in which cells lose their epithelial characteristics and acquire mesenchymal phenotypes. EMT has been associated with stemness, migration, metastasis, and resistance to tumor treatment [1]. Metabolic reprogramming leads to EMT progression and the development of aggressive tumor phenotypes [2]. Cancer cells may acquire cancer stem cell (CSC)-like properties through metabolic reprogramming [3,4]. Cancer cell metabolism depends on a heterogeneous tumor microenvironment and is influenced by the development of the vasculature and blood flow, oxygen concentration, and nutrient energy supply, and it requires the regulatory interplay between multiple oncogenes, transcription factors (TFs), growth factors, and reactive oxygen species (ROS) [5]. The development of the glycolytic phenotype plays a role in the development of tumor aggressiveness, including mitochondrial dysfunction and an acidic tumor microenvironment [6]. Additionally, an altered metabolic microenvironment may result in tumor metastasis. Although several studies on the EMT have been conducted, the clinical implications of metabolic reprogramming for the development of distinct EMT states (high/low) remain elusive. Tumors that undergo metabolic reprogramming are associated with a poor patient prognosis in some cancer types [7], and studies have shown that metabolic reprogramming of tumor cells may affect the tumor immune microenvironment [8]. Importantly, the reprogrammed immune microenvironment influences the immune responses to anti-cancer treatment [9]. Therefore, it is crucial to understand the mechanisms for metabolic reprogramming underlying the EMT states and the impact on patient survival. We aimed to perform a thorough assessment of the connection between seven metabolic signatures [7] and clinical prognostic indicators with EMT states in various cancer types. In particular, our objective was to focus on the common or distinct molecular features mediating the EMT states and to assess their clinical relevance. In view of this goal, we focused on investigating predictive drug targets for the high EMT state associated with energy metabolism in cancers. Understanding these EMT metabolic reprogramming-related markers and pathways may provide insights into unique metabolism-associated features in the EMT states and their intervention using novel drug targets.

## 2. Materials and Methods

### 2.1. Datasets

The normalized expression dataset for mRNA (version 2016.8.16; Platform: IlluminaHiSeq_RNASeqV2, Illumina, San Diego, CA, USA) for 31 cancer types, including eight pooled sets (SARC, kidney renal clear cell carcinoma (KIRC), bladder urothelial carcinoma (BLCA), brain low-grade glioma (LGG), stomach adenocarcinoma (STAD), uveal melanoma (UVM), head and neck squamous cell carcinoma (HNSC), and acute myeloid leukemia (LAML), was obtained from Broad GDAC Firehose (https://gdac.broadinstitute.org/, accessed on 01 August 2020). We used the transcriptome dataset of gastric cancer (Y497, GSE84437, https://www.ncbi.nlm.nih.gov/geo/, accessed on 15 February 2021) as a validation cohort.

### 2.2. EMT and Metabolic Reprogramming Signature Analysis

To assess EMT activity for metabolic reprogramming, we used RNA sequencing (RNA-seq) data for 31 types of cancer. We obtained the EMT and ROS signatures from MSigDB (http://software.broadinstitute.org/gsea/msigdb, accessed on 03 August 2020). In the pre-processing step for mRNA expression, genes with an RNA-Seq by expectation-maximization (RSEM) value of <1 in >50% of the samples were removed. Log_2_-transformed RNA-seq data were used. The activities of the pathways for each sample were obtained using single-sample gene set enrichment with the R package GSVA [10]. We obtained the metabolic signature gene set that was reported in a previous study [7], and this included genes for amino acid metabolism (348 genes), carbohydrate metabolism (286 genes), integrated energy metabolism (110 genes), lipid metabolism (766 genes), nucleotide metabolism (90 genes), tricarboxylic acid cycle (TCA cycle, 148 genes), and vitamin cofactor metabolism (168 genes). To assess the significance of the scores, we estimated *p*-values by generating the background distribution using permutation of the expression profiles (10,000,000 times).

### 2.3. Immune Cell Profiling Analysis

For immune cell profiling from the RNA-seq data across 31 cancer types, we used the CIBERSORT [11] and xCell [12] tools.

### 2.4. Survival Probability Analysis and Functional Protein–Protein Interaction Networks

We used the R package “survival” [13] to perform the overall survival analysis and generate Kaplan-Meier survival plots. The log-rank test was used to compare the survival distributions at a significance level of *p* < 0.05. Analyses were conducted using R version 3.5.1 (July 2018, The Comprehensive R Archive Network, open source). We performed two-sided statistical tests; *p*-values < 0.05 indicate statistical significance.

A functional protein association network was constructed for significant differentially expressed genes (DEGs) using the STRING tool with high confidence and was extended by adding nodes that were connected to the initial network [14].

### 2.5. Prediction of Drug Targets

We performed a drug prediction analysis using genomics for drug sensitivity in cancer (GDSC) [15] data for patients with high energy with high EMT in four cancer types (uveal melanoma (UVM), stomach adenocarcinoma (STAD), head and neck squamous cell carcinoma (HNSC), and acute myeloid leukemia (LAML)). We performed a DEG analysis for high- and low-energy samples in the high EMT group using the R package “limma” [16]. The DEGs were used to identify drug targets. The upregulated genes were plugged into GDSC to predict drugs of interest. DeSigN (http://design.cancerresearch.my, accessed on 15 February 2021) drug repositioning was used to identify drug targets in the high energy metabolic sample with four cancer types (UVM, STAD, LAML, and HNSC) in an unbiased manner [17].

### 2.6. Gene Ontology and TF Analysis

Gene Ontology (GO) analysis was conducted to identify canonical biological pathways using METASCAPE [18], with DEG (false discovery rate (FDR) < 0.01). To identify the master regulatory TFs, we performed a TF analysis using iRegulon and the iRegulon database (version 2015.02.12) [19], which pairs motifs and chromatin immunoprecipitation sequencing (ChIP-seq) tracks to identify the TFs that regulate gene networks. In brief, Cytoscape networks were generated by using the list of upregulated DEGs. The nodes (genes) were provided as variables to iRegulon and analyzed using the following default options: (1) motif collection (10-kb region, 9713 position-weight matrices); (2) track collection (1120 ChIP-seq tracks of ENCODE uniform signals); (3) putative regulatory region (20 kb centered around the transcription start site (TSS)); (4) motif rankings database (20-kb region centered around the TSS; 7 species), and (5) track of rankings database (20-kb region centered around the TSS; ChIP-seq derived). We unselected the TF targets that showed low correlation (enrichment score threshold 3.0; maximum FDR on motif similarity, FDR = 0.001).

## 3. Results

### 3.1. Metabolic Reprogramming in Different EMT Activities across 31 Cancer Types

**Figure 1:**
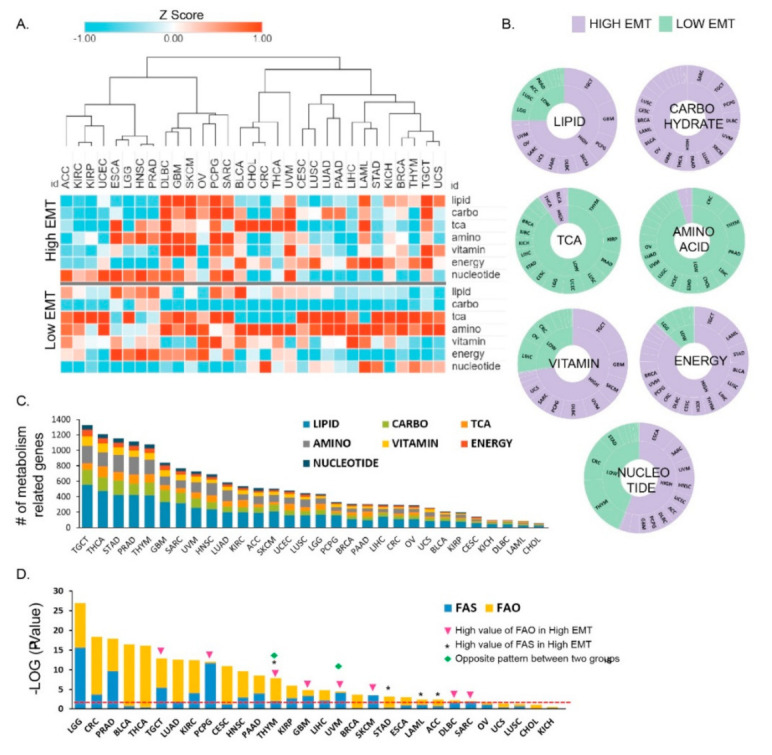
Distinct metabolic reprogramming in different epithelial–mesenchymal transition (EMT) activity/status across 31 cancer types. (**A**) Enrichment of metabolic signatures in high EMT samples showing positive and low EMT activity (high EMT) and in those samples showing negative EMT activity (low EMT). Thirty–one cohorts of tumor samples from The Cancer Genome Atlas (TCGA) were used to determine the EMT activity. The terms indicated are as follows: lipid, lipid signature; carbo, carbohydrate signature; TCA, tricarboxylic acid (TCA) signature; amino, amino acid signature; vitamin, vitamin signature; energy, integrated energy signature. A high *Z*–score indicates high activity in the corresponding metabolic reprogramming signatures. (**B**) Pie chart for seven metabolic signatures, showing the fraction of cancer types that classify as high and low EMT and show distinct EMT activity (high: purple; low: mint green). Lipid, carbohydrate, and energy had larger fractions (80 to 90%) in high EMT. TCA and amino acid showed larger fractions (80 to 90%) in low EMT. (**C**) The bar graph shows the number of differentially expressed metabolic signature genes between high and low EMT. The testicular germ cell tumor (TGCT) cancer type was found to be top-ranked. (**D**) Bar graph depicting the significant *p-values* for fatty acid synthesis (FAS) and fatty acid oxidation (FAO) that were identified between high and low EMT. * indicates that FAO is higher in low EMT than it is in high EMT. Triangles indicate samples in which FAS is higher in high EMT than in low EMT. Diamonds indicate the samples for one cancer type that have the opposite result for lipid metabolism between high and low EMT.

To explore transcriptional metabolic reprogramming based on EMT activity, we analyzed the RNA-seq data of 31 cancer types from The Cancer Genome Atlas (TCGA) and examined them for information regarding their EMT activity (Table S1). To analyze the transcriptome signatures in metabolic reprogramming, we examined the gene set for seven metabolic signatures based on the reactome annotation [7,20], including amino acid metabolism (348 genes), carbohydrate metabolism (286 genes), integrated energy metabolism (110 genes), lipid metabolism (766 genes), nucleotide metabolism (90 genes), TCA cycle (148 genes), and vitamin cofactor metabolism (168 genes) (Table S2). To analyze the different EMT activities in each tumor, we used the single-sample gene set variation enrichment analysis approach [10]. We split the samples into two groups for each cancer type: the high EMT (positive EMT enrichment score) and the low EMT (negative EMT enrichment score) samples. Next, we analyzed the enrichment scores for seven metabolic signatures based on the EMT states across cancer types. Our results showed that the metabolic reprogramming activity in the high and low EMT groups varied across cancer types. Of note, among the seven metabolic signatures, carbohydrate metabolism (*p* = 1.22 × 10^−16^) and energy metabolism were significantly enriched (*p* = 1.22 × 10^−16^) in samples with high EMT relative to those with low EMT. However, amino acid metabolism (*p* = 1.22 × 10^−16^), TCA cycle (*p* = 1.22 × 10^−16^), and lipid metabolism (*p* = 0.00044) were significantly enriched in cancers with low EMT (Figure S1). Among a total of 9452 samples, 58.57% showed the presence of high carbohydrate levels and high EMT activity relative to low EMT activity samples (Figure 1A).

Taken together, our results show that a distinct metabolic activity is associated with different EMT states. Interestingly, in several cancer types, including lymphoid neoplasm diffuse large B cell lymphoma (DLBC), skin cutaneous melanoma (SKCM), pheochromocytoma and paraganglioma (PCPG), UVM, and testicular germ cell tumor (TGCT), the expression of the seven metabolic signatures was increased (*p* < 0.05) and showed high EMT activity (Figure 1A). In contrast, in a few other cancer types, only one metabolic signature was increased and showed high EMT activity; for example, adrenocortical carcinoma (ACC) displayed high nucleotide levels with high EMT activity, whereas STAD [21] and liver hepatocellular carcinoma (LIHC) showed high-energy reprogramming in high EMT. Approximately 80 to 90% of cancer types showed significantly greater enrichment for the TCA and amino acid pathways in the low EMT group than in the high EMT group (Figure 1A and Figure S1). Overall, our analyses showed that the seven metabolic signatures might be regulated distinctively in individual cancer types by EMT state. Notably, carbohydrate and energy metabolism were significantly enriched in high EMT tumors. The number of differentially expressed metabolic signature genes depended on the specific cancer type, with TGCT being the top-ranked tumor (*n* = 1328; Figure 1C). *GAPDH* expression showed the highest fold-change (FC), and *STAR* had the lowest fold-change among 30 cancer types (Table S3). *GAPDH* promotes cancer growth and metastasis through upregulation of *SNAIL* expression [22] in multiple tumor types and is associated with tumor proliferation, metastasis, and an overall aggressive tumor phenotype [23]. Tumor cells show dysregulated lipid metabolism based on their high lipogenic and low lipolytic capacity and upregulation of bioactive lipid production that promotes EMT processes [2]. Lipid metabolism was classified based on two mechanisms, namely fatty acid synthesis (FAS) and fatty acid oxidation (FAO), and was significantly enriched (93.33%; 28 cancer types) in most low EMT tumors (Figure S1). Thymoma (THYM) and UVM showed different expression patterns between FAS and FAO. We found that 17 cancer types have a significant difference in FAS, and 20 cancer types have a significant difference in FAO (Figure 1D). Brain low-grade glioma (LGG) was top-ranked for both FAS and FAO and showed a significant difference between high versus low EMT (Figure 1D). Collectively, these results suggest that in most cancer types, one or more metabolic reprogramming pathways are increased based on EMT activity. However, in some cancer types, only one metabolic reprogramming pathway may show an increase, especially in high EMT.

The results suggest that metabolic reprogramming is not a simple process involving the migration and invasion of cancer cells, and a change to a more complex tumor microenvironment may lead to the development of aggressive tumors that undergo EMT.

### 3.2. Metabolic Reprogramming Is Correlated with EMT Activity that Affects the Tumor Microenvironment

**Figure 2:**
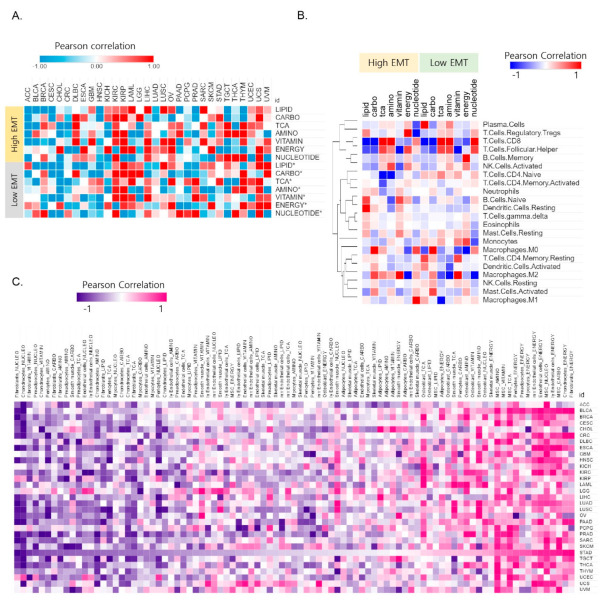
The correlation between metabolic reprogramming and EMT activity affects the tumor microenvironment. (**A**) Correlation between the signature for reactive oxygen species (ROS) and 7 metabolic signatures in the high and low EMT groups. (**B**) Heat map for the Pearson correlation coefficients between the 22 major immune cell signatures and 7 metabolic signatures showing distinct EMT activity. The information for the 22 immune cell types was obtained using CIBERSORT. (**C**) Heat map of the Pearson correlation coefficients between 13 stromal signatures and 7 metabolic signatures in high EMT samples. The information for the 13 stromal cell types was obtained using the xCell tool. Stomach adenocarcinoma (STAD) was highly correlated between stromal cell types and energy metabolism in 31 cancer types.

Metabolic reprogramming in the tumor microenvironment determines the immune response to cancer [9,24]. Tumor metabolic reprogramming affects the function of macrophages, T cells, and myeloid-derived suppressor cells, among other immune cells [25]. A recent study showed that EMT is negatively associated with the response to immunotherapy [26]. Furthermore, energy metabolism wields the fate and function of tumor myeloid-derived suppressor cells [27]. Therefore, we examined the relationship between the seven major metabolic signatures and the characteristics of the tumor microenvironment in specific cancer types. To this end, we first assessed the immune cell characteristics in different EMT states. By using CIBERSORT [11], we scored 22 immune cell types for their relative abundance in tumor samples with different EMT activity. Next, we evaluated the correlation between the 22 immune cell signatures and seven metabolic signatures. The high EMT samples for the 31 cancer types showed a significant correlation with macrophages (M2) and regulatory T cells (T_reg_) (*p* < 0.05) (Figure 2B). CD8 was highly correlated with TCA and amino acids in low EMT, and T_reg_ was correlated with integrated energy metabolism in high EMT. High carbohydrate metabolism was correlated with macrophages (M2).

The metabolic reprogramming of T_reg_ mainly inhibits glycolysis and promotes FAO and OXPHOS (oxidative phosphorylation), which promote cell proliferation, differentiation, and immune function [28]. Metabolic reprogramming that modulates T cell ROS generation and antioxidant capacity [29] showed different correlation patterns in high EMT and low EMT (Figure 2A). For example, ROS are highly correlated (*R* = 0.87) with carbohydrates for STAD in high EMT but have a high correlation value (*R* = 0.85) with TCA in low EMT. ROS plays a crucial role in maintaining and promoting the tumor phenotype via the regulation of cellular metabolism processes and oncogenic signaling pathways [30]. Overall, these results show that metabolic reprogramming is intrinsically coupled with immune cell function via EMT activity.

Furthermore, we explored the correlations between stromal cell types and metabolic reprogramming of the immune microenvironment variables in the context of cancer types. Under certain conditions, tumor cells can convert these reactive stromal cells further and transition them into tumor-associated stromal cells (TASCs) [31]. TASCs, as key contributors to the tumor microenvironment, can affect tumor progression and tumor-associated fibroblasts. We used xCell [12] to gain information for stromal cell types (adipocytes, chondrocytes, endothelial cells, fibroblasts, mesenchymal stem cells, myocytes, osteoblasts, pericytes, preadipocytes, skeletal muscle cells, smooth muscle cells, lymphatic endothelial cells, and microvascular endothelial cells). We found that stromal cells are highly correlated with energy metabolism in high EMT. STAD is a top-ranked cancer type that shows a high correlation (*p* < 0.05) among the 31 cancer types (Figure S2). We identified distinct patterns of correlation between stromal cells and the seven metabolic reprogramming pathways by sorting the correlation values in descending order based on their association with STAD [21] (Figure 2C). The correlation between stromal cell types and metabolic reprogramming was positive/negative depending on the type of cancer (Figure 2C). Collectively, these results indicate the potential therapeutic benefits of tumor microenvironment-mediated therapy in energy metabolism reprogramming.

### 3.3. Distinct Metabolic Reprogramming of EMT Activity Is Associated with Prognosis in a Specific Cancer Type

**Figure 3:**
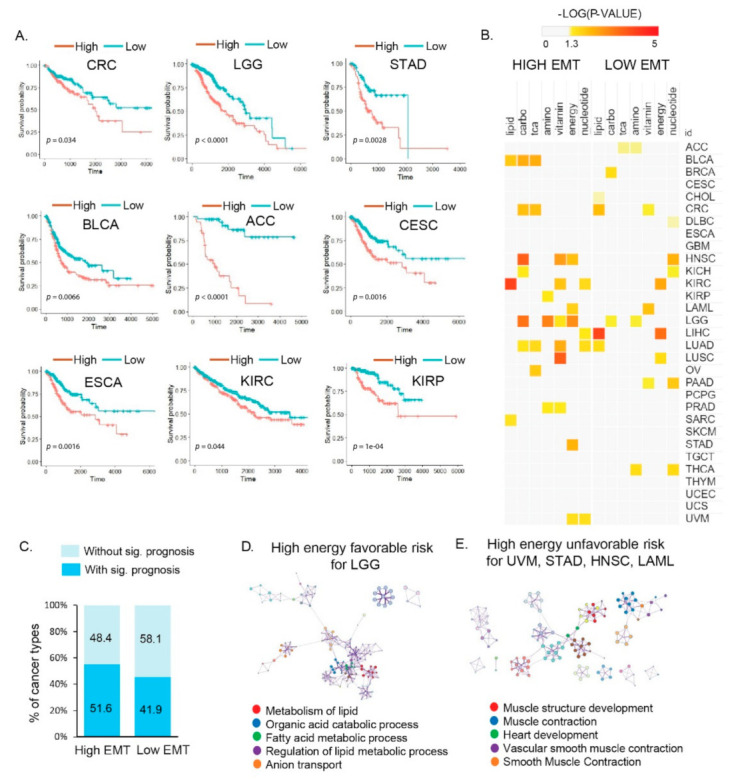
Molecular and clinical features of different metabolic reprogramming processes affect prognosis according to EMT activity in multiple cancer types. (**A**) Kaplan–Meier plots showing the overall survival rates for the high and low EMT groups. The *p*-values were analyzed using the log–rank test and adjusted by Bonferroni correction. Nine cancer types (CRC, LGG, STAD, BLCA, ACC, CESC, ESCA, KIRC, and KIRP) showed significantly different prognoses. Abbreviations: CRC, colon adenocarcinoma; LGG, brain low–grade glioma; STAD, stomach adenocarcinoma; BLCA, bladder urothelial carcinoma; ACC, adrenocortical carcinoma; CESC, cervical squamous cell carcinoma and endocervical adenocarcinoma; ESCA, esophageal carcinoma; KIRC, kidney renal clear cell carcinoma; KIRP, kidney renal papillary cell carcinoma. (**B**) Clinical association of metabolic reprogramming with the overall survival times in patients. The color scale indicates the statistical significance (*p* < 0.05, yellow). (**C**) Percentages of cancer types with and without significant prognosis based on high and low EMT activity, respectively. (**D**) Top five Gene Ontology clusters for high energy and favorable patient outcomes with high EMT in LGG (left). (**E**) Top five Gene Ontology clusters for high energy and unfavorable patient outcomes with high EMT in uveal melanoma (UVM), STAD, head and neck squamous cell carcinoma (HNSC), and acute myeloid leukemia (LAML) (right).

We investigated the effects of different types of metabolic reprogramming on cancer prognosis. Studies have shown that high metabolic reprogramming in specific cancer types has a poor prognosis [7]. Thus, we comprehensively examined the relationships between the different metabolic reprogramming signatures and the clinical outcomes in 31 cancer types. We compared the survival rates for patients in the high and low EMT groups (Figure S3). Nine cancer types showed a significantly different prognosis (Figure 3A). Sixteen cancer types with high EMT, including UVM, STAD, SARC, BLCA, LIHC, LUAD, colon adenocarcinoma (CRC), HNSC, KICH, LGG, LAML, KIRC, kidney renal papillary cell carcinoma (KIRP), LUSC, OV, and PRAD, showed significant (*p* < 0.05) results for one or more types of metabolic reprogramming (Figure 3B, Figure S4) in high EMT. Sixteen (51.9%) and thirteen (41.9%) cancer types showed significant results based on high and low EMT activity, respectively (Figure 3C). In the SARC, KIRC, and BLCA cancer types, lipid activity showed a trend opposite to the survival rate in the high EMT group. High lipid metabolism was associated with a better prognosis in KIRC (*p* < 0.0001) and SARC (*p* = 0.036). However, BLCA showed a favorable survival rate for low lipid metabolism (*p* = 0.014) (Figure S4). High energy metabolism (*p* = 0.0017) was associated with a better prognosis for LGG with high EMT. Patients with favorable clinical outcomes had low energy metabolic activity in UVM, STAD, HNSC, and LAML, (Figure S4), and several studies have shown that a poor prognosis is observed in patients with high energy metabolic reprogramming [32].

In the low EMT group, cancer types showed significantly different survival probabilities (Figure S5). Low EMT LIHC cancer showed a favorable prognosis with high lipid metabolic reprogramming. Among the lipid metabolism-related signatures, FAO and FAS were differentially enriched according to the EMT state. Since anabolic lipid metabolism (i.e., FAS) is required for cancer cell proliferation, low EMT LIHC that showed low FAS had a good prognosis, linking tumor metabolic reprogramming and clinical outcomes.

We performed a GO analysis to obtain functional insights into metabolic reprogramming in different EMT states that were related to the clinical outcomes. In the high EMT groups of SARC and KIRC, biological pathways for high lipid metabolism that were related to favorable outcomes showed an enrichment for genes related to the organic acid catabolic process; carbon metabolism; valine, leucine, and isoleucine degradation; cofactor metabolic process; and cellular amino acid metabolic processes (FDR < 0.001; Figure S4, Table S5). In BLCA, the biological pathways related to favorable outcomes were “Metabolism of RNA”, “Response to type I interferon”, and “TNFR2 non-canonical NF-kB pathway”; similarly, in LGG, the “metabolism of lipid”, “organic acid catabolic process”, and “fatty acid metabolic process” were enriched (Figure 3D, Table S5). In the high-energy groups for UVM, STAD, HNSC, and LAML, the biological pathways for high EMT related to poor patient outcomes were enriched for genes related to “muscle structure development”, “muscle contraction”, “heart development”, “vascular smooth muscle contraction”, and “smooth muscle contraction” (Figure 3E). The association of survival patterns with metabolic reprogramming indicates the presence of cancer type-specific functions of EMT states. Thus, our findings indicate the potential use of metabolic reprogramming by EMT activity as a predictor for patient outcomes.

### 3.4. Vulnerabilities of Integrated Energy Metabolic Reprogramming for Cancer Therapy

**Figure 4:**
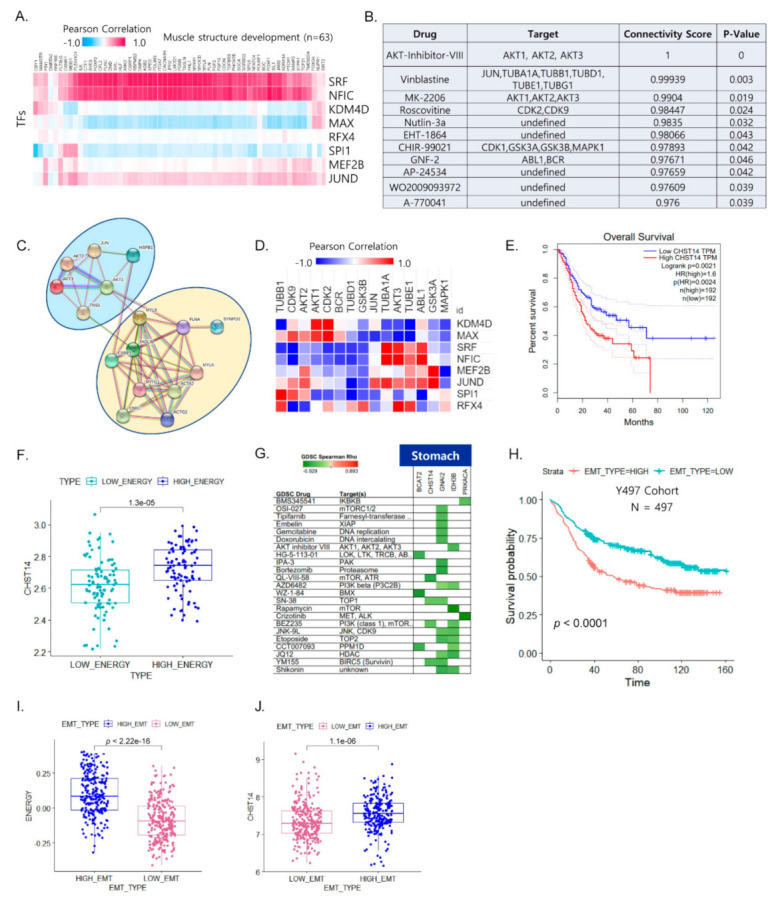
Integrated energy metabolic reprogramming is correlated with drug sensitivity. (**A**) Heat map of the Pearson correlation coefficients between transcription factors (TFs) and muscle structure development genes, with the top–ranked biological pathways for four cancer types (UVM, STAD, LAML, and HNSC) with high EMT. (**B**) Predicted compounds and target genes for four cancer types (UVM, STAD, LAML, and HNSC) that were obtained using DeSigN. (**C**) Protein–protein interaction network between the top 21 predicted drug target genes and muscle–related genes for four cancer types (UVM, STAD, LAML, and HNSC). (**D**) Heat map of the Pearson correlation between the predicted drug target genes and TFs for four cancer types (UVM, STAD, LAML, and HNSC) (red: positive correlation; blue: negative correlation). (**E**) Kaplan–Meier plot showing the recurrence–free survival rates for high and low *CHST14* expression in STAD for the TCGA cohort. (**F**) Box plot of *CHST14* expression for distinct energy activity in STAD. (**G**) Functional genomics for drug sensitivity in cancer (GDSC) and target genes for STAD. Green (negative correlation) indicates high drug sensitivity. (**H**) Kaplan–Meier plot showing overall survival rates for high and low EMT expression in gastric cancer of Y497 cohort. (**I**) Box plot of energy signature for distinct EMT states in Y497 cohort. (**J**) Box plot of *CHST14* expression for distinct EMT states in Y497 cohort.

To explore the specific transcriptional regulatory network for the distinct metabolic signatures, we investigated the regulatory association between genes that belong to the prognosis-associated biological pathways in metabolic reprogramming. Our results showed that four cancer types (i.e., UVM, STAD, HNSC, and LAML) were associated with TFs (FDR < 0.0001) that may regulate muscle structure development. Indeed, SRF and NFIC were identified as the top positively correlated TFs, which are known to control muscle structure development and myogenesis genes (Figure 4A). To examine the therapeutic vulnerability of metabolic reprogramming in high EMT cancer, we used DeSigN (http://design.cancerresearch.my, accessed on 15 February 2020) to identify drug targets for the high energy metabolic reprogramming samples using four cancer types (UVM, STAD, LAML, and HNSC) in an unbiased manner. The DeSigN drug repositioning analysis [17] identified potential compounds/inhibitors that are capable of targeting the high energy signature (FDR < 0.0001) (Figure 4B). We found an enrichment for compounds associated with high energy in at least four cancer types with high EMT. Five compounds, i.e., AKT-Inhibitor-VIII, vinblastine, MK-2206, roscovitine, and nutlin-3a, were significantly enriched for high energy in four cancer types (UVM, STAD, LAML, and HNSC) with high EMT and inhibited tumor aggressiveness-related tumorigenicity. Among these, *AKT1*, a target of AKT-inhibitor-VIII, was more closely linked with other muscle-related genes, indicating its functional importance as a therapeutic target (Figure 4C). AKT1, which regulates many cellular processes in cancers, including metabolism, proliferation [33], cell survival, growth, and angiogenesis, may serve as a potential biomarker for the prediction of prognosis for patients and the identification of high-risk cases [34].

Next, we assessed the correlation between TFs and the predicted drug target genes. *ABL1* was positively correlated with five TFs (Figure 4D), suggesting that the FDA-approved anti-cancer therapeutic drug imatinib could be repurposed for high-EMT tumors with energy metabolic reprogramming in poor prognostic outcomes. We further examined stomach samples of patients with high energy in high EMT. We found five metabolism-related genes (*BCAT2*, *CHST14*, *GNAI2*, *IDH3B*, and *PRKACA*) out of 93 upregulated genes (*p* < 0.05) in high-energy samples. Among the five upregulated metabolic genes in the high-energy samples, the increased expression of *CHST14* was significantly associated with poor prognosis (Figure 4E). We further investigated its clinical relevance using the pooled STAD dataset, which indicated that high energy metabolism was associated with high expression of *CHST14* (Figure 4F). Thus, considering *CHST14* as a legitimate target, we identified candidate drugs (YM155, BEZ235, and SN-38) against *CHST14* for stomach cancer (Figure 4G). We further validated its clinical relevance using an independent GC dataset (Y497 cohort: GSE 84437) that confirmed poor clinical outcome being associated with high EMT state (Figure 4H). Additionally, energy signature was significantly increased in the high EMT state (Figure 4I) and *CHST14* expression was higher in the high EMT group than in the low EMT group (Figure 4J).

In contrast, LGG showed a favorable prognosis (*p* = 0.0017) for high energy with high EMT (Figure S4). We found that the response to type I interferon was enriched in the high-energy group and was associated with favorable patient outcomes (Table S4), suggesting that immune surveillance may be attributed to the outcomes. Taken together, our results outline therapeutically exploitable genomic markers for drug sensitivity that may prove to be useful in future clinical trials for biomarkers in metabolic gene-targeting therapy.

## 4. Discussion

Metabolic reprogramming is an essential pathway for events that mediate malignant transformation, including EMT, that are a hallmark of cancer [35], thus promoting tumor metastasis. The induction of EMT promotes the proliferation of tumor cells from the primary tumor site and enhances the self-renewal ability of tumor cells. EMT can transform tumor cells into CSCs, which may acquire a migratory ability through EMT [36]. Although metabolic reprogramming occurs in some cancers, it is unclear why specific metabolic reprogramming occurs in relation to different EMT states in certain cancer types. Our pan-cancer analysis of metabolic reprogramming from different EMT states indicated that differential metabolic reprogramming frequently occurs in tumors showing high and low EMT states. Furthermore, metabolic reprogramming depends on the presence of cancer hallmarks such as ROS, DNA repair, angiogenesis, and hypoxia and can be associated with the immune cell microenvironment. Cells such as M2 and T_reg_ in the tumor microenvironment change ATP to adenosine, which can suppress the activity of other immune cells in the tumor [37]. Our results showed that the overexpression of energy-related genes was positively correlated with T_reg_ cells in the pooled samples with high EMT samples. Our results are in line with those from previous studies demonstrating that the cellular component of the tumor microenvironment can facilitate the EMT process of malignant cells. In particular, the presence of suppressive or exhausted immune cells, such as M2 and T_reg_ cells, may create an “EMT-permissive state” through TGF-β, which enhances Snail TFs in cancer cells. Furthermore, Snail, a key transcriptional regulator of EMT, regulates glucose flux and facilitates glycolysis and the pentose phosphate pathway, enabling cancer cell survival under metabolic stress.

The prognosis associated with metabolic reprogramming in different cancer types may also have implications for harnessing the vulnerability of high EMT in precision medicine for the treatment of cancer patients. We showed that distinct metabolic reprogramming in different EMT states influences patient prognosis. Moreover, we identified that carbohydrate-, vitamin-, and energy-related genes were highly expressed in the high EMT group in 18 cancer types (ACC, UCEC, DLBC, GBM, SKCM, PCPG, SARC, BLCA, UVM, LUAD, PAAD, LIHC, LAML, BRCA, THYM, TGCT, and UCS). In contrast, TCA and amino acid-related genes were highly expressed in the low EMT group of 28 cancer types (ACC, KIRC, KIRP, UCEC, LGG, DLBC, GBM, SKCM, OV, PCPG, SARA, BLCA, CHOL, CRC, THCA, UVM, CESC, LUSC, LUAD, PAAD, LIHC, LAML, STAD, KICH, BRCA, THYM, TGCT, and UCS). Our results showed that high-energy samples with high EMT states showing energy metabolic reprogramming were closely associated with the development of muscle structure and a muscle-like phenotype. Indeed, these results are in line with those from previous studies showing that tumors with high EMT activity (i.e., EMT or mesenchymal subtype tumors) are associated with elevated expression of smooth muscle cell genes (e.g., MYH11 and ACTA2), resembling muscle cell transcriptome features [38,39]. Typical smooth muscle cell metabolism is compartmentalized, and dual energy-generating metabolic pathways are activated to support muscle contraction and membrane pumps by mitochondrial function and glycolytic ATP production, respectively [40]. Altogether, these factors explain why the muscle-like phenotype and transcription programs are associated with high EMT states of tumor and energy metabolic reprogramming.

The metabolic reprogramming of EMT states is associated with distinct prognostic outcomes in different cancer types. Of note, patients with LGG had better survival probabilities with higher energy activity; however, patients with UVM, STAD, HNSC, and LAML had better survival probabilities with lower energy activities. The prognosis depended on the energy activity of the tissue type in high EMT states. A functional analysis of the genes associated with higher energy activity indicated that the response of type I interferon (IFN-I) plays a central role in driving the antiviral state in non-immune cells and in orchestrating antiviral immune responses [41] in LGG, unlike in other cancer types. In LGG, the signatures for IFN-I were increased in the presence of low levels of energy and were associated with poor patient outcomes. Novel therapeutic strategies that take into consideration the tumor microenvironment may be potentially useful in patients with glioblastoma having low levels of energy and high EMT states. Furthermore, we assessed potential drug targets for four cancers (UVM, STAD, HNSC, and LAML) with high EMT activity using data from a drug sensitivity database. In the four cancer types, *AKT1* was identified as a potential drug target. The overexpression of *AKT1* observed in invasive cancer cells is associated with increased expression of glucose and energy metabolism and is associated with increased glycolysis and EMT in gastric cancer cells [42]. In the four cancer types, AKT and ABL1 were identified as potential drug targets. Intriguingly, the AKT PPI network was closely associated with muscle-related modules, further confirming the biological implications of the identified targets. Furthermore, clinical studies evaluating AKT inhibitors in patients with UVM (ClinicalTrials.gov Identifier: NCT01979523) and HNSC (ClinicalTrials.gov Identifier: NCT01349933) showed early clinical efficacy, indicating the potential validity of our analyses.

In summary, we demonstrated that metabolic reprogramming of different EMT states operates differently depending on the type of cancer. In addition, the activation of energy metabolic reprogramming could affect the prognosis of patients with specific cancer types. Therefore, these results provide valuable insights for the development of precision medicine to treat aggressive tumors. Additional research is required to evaluate drugs for cancers associated with the high energy of high EMT states. In the future, candidate target drugs must be validated in order to conduct efficacy studies using in vivo and in vitro assays, such as analyses using cell lines or patient-derived xenograft models.

## 5. Conclusions

We successfully demonstrated that metabolic reprogramming based on EMT activity differentially depends on the cancer type. Moreover, energy metabolic reprogramming may affect the prognosis of patients with different cancer types with high EMT (UVM, STAD, HNSC, LAML, and LGG). Our results provide valuable insights for the development of precision medical therapy to treat recalcitrant tumors.

## Data Availability

The results shown here are in part based upon data generated by the TCGA Research Network: http://cancergenome.nih.gov.

## Supplementary Materials

The following are available online at https://www.mdpi.com/article/10.3390/cancers13081778/s1, Figure S1: Boxplot demonstrating pathway enrichment scores of each corresponding pathway, Figure S2: Heatmap of pearson correlation between 7 metabolic reprogramming and stromal immune cell types, Figure S3: Kaplan–Meier plots of overall survival for the EMT activity in 31 cancer types, Figure S4: Kaplan–Meier plots of overall survival for the metabolic reprogramming with high EMT group in 16 cancer types (UVM, STAD, SARC, BLCA, LIHC, LUAD, CRC, HNSC, KICH, LGG, LAML, KIRC, KIRP, LUSC, OV and PRAD), Figure S5: Kaplan–Meier plots of overall survival for the metabolic reprogramming with high EMT group in 12 cancer types (STAD, LIHC, LGG, BRCA, CRC, HNSC, KICH, LAML, KIRC, LUAD, LUSC, PAAD and THCA), Table S1: List of 31 TCGA cancer types, Table S2: List of genes involved in 7 metabolic signatures, Table S3: List of significant signature genes involved in metabolic reprogramming across cancer types, Table S4: List of significant gene ontology (GO) terms enriched in high energy group and low energy group, Table S5: List of significant gene ontology (GO) terms enriched in high lipid group and low lipid group.
